# Minimally invasive converted to open versus upfront open surgeries for rectal cancer: a retrospective cohort study

**DOI:** 10.1007/s00464-025-11958-0

**Published:** 2025-07-08

**Authors:** Rachel Ma, George Wu, Sriya Chilla, Paola Solis-Pazmino, Moshe Barnajian, Yosef Nasseri

**Affiliations:** 1 Surgery Group Los Angeles, Los Angeles, CA USA; 2 Surgery Department, Santa Casa de Porto Alegre, Porto Alegre, RS Brazil; 3 Cedars Sinai Medical Center, Los Angeles, CA USA; 4 8635 W 3Rd St Suite 880, Los Angeles, CA 90048 USA

**Keywords:** American College of Surgeons National Surgical Quality Improvement Program (ACS-NSQIP), Rectal neoplasms, Surgical conversion, Proctectomy, Retrospective study, Propensity score

## Abstract

**Background:**

Minimally invasive surgery (MIS) has significantly better short-term outcomes than open surgery. However, it is unclear whether MIS converted (MISC) to open for rectal cancer carries any benefits compared to open as no prior study has compared these two surgical approaches for rectal cancer. This study compares short-term postoperative outcomes between MISC and upfront open surgery.

**Methods:**

This is a retrospective cohort study on elective surgeries for rectal cancer from 2016 and 2022 from the American College of Surgeons National Surgical Quality Improvement Program (ACS-NSQIP) database. Patients were divided into the MISC group and the Open group and balanced using propensity-score matching. 30-day complication, unplanned readmission, and mortality rates and length of stays were compared with Student’s t-test and regression models.

**Results:**

The Open group included 2551 (86.3%) patients, and the MISC group included 406 (13.7%) patients (80.5% laparoscopy, 19.5% robotic). Propensity-score matching yielded 400 patients in each group. On univariate analysis, the groups had comparable overall complications, unplanned readmission, and 30-day mortality rates, but the MISC group had a lower median length of stay (6 days vs. 7 days, *p* = 0.015). On regression analysis, the MISC was not associated with an increased risk for overall complications (OR = 1.04, 95%CI 0.843–1.280; *p* = 0.716), unplanned readmission (OR = 1.230, 95%CI 0.939–1.620; *p* = 0.132), and 30-day mortality (OR = 1.750 95%CI 0.648–4750; *p* = 0.269). However, MISC was significantly associated with a shorter length of stay (OR = − 0.869, 95%CI (−)1.609 − (−)0.129; *p* = 0.021).

**Conclusion:**

Patients undergoing planned open proctectomy and those undergoing MISC to open proctectomy exhibit similar 30-day postoperative outcomes. As completed MIS offers advantages over open surgeries, MIS should be attempted as the default surgical approach for rectal cancer patients, even if conversion to open surgery is needed.

**Supplementary Information:**

The online version contains supplementary material available at 10.1007/s00464-025-11958-0.

Rectal cancer continues to be a significant health concern, with the American Cancer Society estimating 46,220 new cases in the US in 2024, making it the third most common in both men and women [1, 2]. Notably, the prevalence of this cancer in younger patients is trending upward [3]. This trend underscores the importance of effective and safe surgical intervention, especially considering the 5-year relative survival for rectal cancer drops steeply from 90% for localized disease to 18% for metastatic disease [1].

Recent trends indicate a significant shift toward minimally invasive approaches for rectal cancer, particularly with the rapid adoption of robotic surgery, which represented 5.8% of rectal cancer surgeries in 2010 to 28.4% in 2019 [4, 5]. As a result, the growth of robotic surgery has led to fewer open surgeries overall, as well as fewer conversions to open surgery compared to laparoscopic approaches [6].

While minimally invasive surgery (MIS) has become more prevalent, unplanned conversion to open surgery is still sometimes necessary; conversion occurs in between 12 and 30% of laparoscopic rectal resections [7–10]. Risk factors for conversion include high body mass index, prior abdominal surgery, and surgeon experience [9, 11]. Conversion has been shown to have an adverse effect on short-term patient outcomes and is associated with increased morbidity, increased mortality, and prolonged recovery when compared to minimally invasive surgery completed without the need for conversion [10, 11].

Although MIShas become the preferred approach for many rectal cancer cases, there are specific scenarios where upfront open surgery is recommended. These include patients with prior abdominal operations, anatomical complexity, or cases requiring complex multivisceral resection. Additionally, in emergency settings such as bowel obstruction or perforation, open surgery may be necessary to ensure timely and safe intervention [12–14]**.**

While it is well established that MIS confers improved short-term outcomes compared to open surgery, there are limited data comparing outcomes between upfront laparotomies and cases starting with an MIS approach and consequently converted to open. There are a few publications comparing these approaches but not for complex pelvic surgery or rectal cancer [9, 15]. Therefore, in this study, we aimed to explore if initial MIS attempts, even when conversion is eventually required, provide any benefit in short-term outcomes relative to planned open rectal surgeries. We used the ACS-NSQIP, a large multi-institutional database to evaluate short-term postoperative outcomes following upfront open surgeries and MIS converted to open surgery in patients undergoing surgery for rectal cancer. This cohort study has been reported in line with the STROCSS guidelines [16]**.**

## Materials and methods

### Study design

This is a retrospective cohort analysis of the American College of Surgeons National Surgical Quality Improvement Program (NSQIP) database that included all patients with TNM stage I-III rectal cancer patients who underwent elective resection between 2016 and 2022. The NSQIP database is a robust database that, after written patient consent, tracks and enhances surgical care quality by collecting clinical and surgical data, including with 30-day outcomes, from around 700 U.S. hospitals. It includes over 150 variables on preoperative risks, intraoperative details, and postoperative outcomes for major surgeries. Trained Surgical Clinical Reviewers gather data through chart reviews and operating room logs, with each hospital appointing a surgeon to oversee quality improvement. De-identified data are shared for national benchmarking, supported by rigorous training to ensure accuracy. The use of deidentified retrospective data eliminates the need for ethical approval. NSQIP is a well-validated and widely respected resource in surgical care [17].

### Data review and selection criteria

**Fig. 1:**
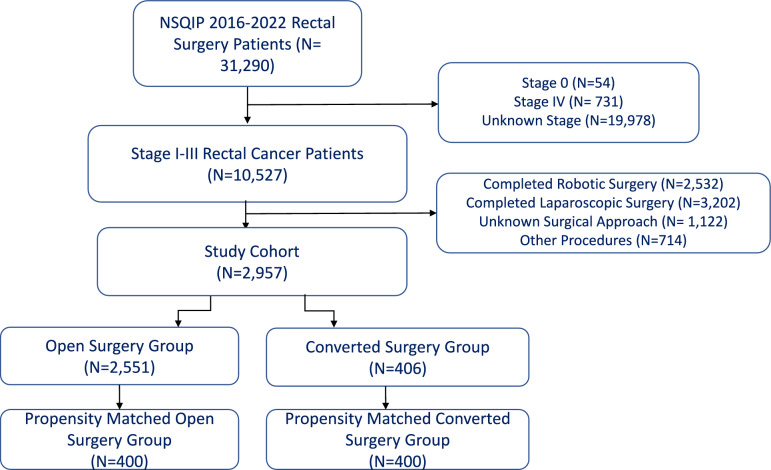
Inclusion and exclusion criteria flowchart

Patients with completed MIS surgery or unknown procedures were excluded. Patients were then classified into two groups, patients operated with an upfront open surgical approach (Open Surgery group) and those operated initially with a minimally invasive approach but were subsequently converted to an open approach (MISC group) (Fig. 1). Conversion to open was defined as an unplanned intraoperative switch from a MIS approach, such as laparoscopic or robotic, to an open approach.

### Propensity-score matching

The two groups were matched for potential confounders using propensity-score matching with a 1:1 ratio and a caliper width of 0.2. Adequate balance between the groups was confirmed by a standardized mean difference (SMD) of less than 0.1. The matching criteria included demographics, such as age, sex, race, and BMI, and comorbidities, including the presence of ascites, dyspnea, history of chronic obstructive pulmonary disease (COPD), preoperative dialysis, preoperative transfusion, preoperative radiation, preoperative sepsis, and preoperative ventilator dependence.

### Data collected

Data were collected on patient demographics, comorbidities, and surgical characteristics. Demographics included age, sex, BMI, and race. Comorbidities included ascites, bleeding disorders, chemotherapy smoking status, diabetes, dyspnea, history of chronic heart failure (CHF), history of COPD, hypertension, dialysis, transfusion, radiation, sepsis, steroid use, and ventilator dependence. Patient and surgical characteristics were operative approach, principle procedure, and tumor location.

The primary outcome was defined as 30-day postoperative complication rate, whereas secondary outcomes included 30-day mortality, 30-day unplanned readmission, and postoperative length of stay.

### Statistical analysis

Statistical analyses were conducted using EZR (version 1.55, from Jichi Medical University Saitama Medical Center)15 and R software (version 4.1.2, from the R Foundation for Statistical Computing) [18]. Continuous variables were reported as the median and interquartile range when not normally distributed. Comparisons of continuous variables were made using either the Student’s t-test or the Mann–Whitney U test, while categorical variables were presented as counts and percentages and analyzed using the χ^2^ test. Logistic and linear regression analyses were performed to identify the predictors of major postoperative complications. Results are presented as an odds ratio (OR) with a 95% confidence interval (CI). A two-sided p value of less than 0.05 was deemed statistically significant.

## Results

From 31,290 patients that underwent rectal resections from cancer, 2,957 cases were included in the final cohort. The flowchart of patients included in the study is shown in Fig. 1. In our final cohort, 2551 patients (86.3%) underwent planned open surgeries (Open Surgery group) and 406 (13.7%) underwent minimally invasive surgery that had an unplanned conversion to open surgery (MISC group). The MISC group and Open Surgery group had similar median ages [63.5 (range 26–90) vs. 63 (range 22–90); *p* = 0.539] and race distributions (*p* = 0.510). BMI was significantly higher in the MISC group compared to the open surgery group [29.4 (range 15.9–62.7) vs. 27.3 (range 14.4–64.4); *p* < 0.001], and the MISC group also had a significantly higher proportion of male patients (69.5% vs. 60.1%, *p* < 0.001). In addition, the MISC group had higher rates of patients suffering from hypertension requiring medication (51.7% vs. 43.7%, *p* = 0.003) and lower rates of smoking (14.8% vs. 19.7%, *p* = 0.017). The two groups also had a different distribution of tumor locations; the MISC group had a significantly lower proportion of patients with tumors located in the lower third of the rectum (42.1% vs. 48.8%, *p* < 0.001) and lower rates of abdominoperineal resection (68.2% vs. 76.3%, *p* = 0.001) (Table S1). In the postoperative outcomes univariate analysis, the MISC group had a statistically higher number of nodes harvested (16.0 vs. 15.0, *p* = 0.007). However, this may not be clinically relevant. There was also a trend toward higher organ space SSI in the MISC group, but this was not statistically significant (10.8% vs. 8.0%, *p* = 0.054). For detailed a comparison of outcomes for the unmatched cohort, please refer to Supplementary Table S2.

### Analysis of the matched cohort

Propensity-score matching yielded 400 patients in each group, and all baseline demographics and comorbidities were similar between the two cohorts (Table 1). No significant difference was found between the MISC and Open Surgery group in the rate of postoperative complications (48.8% vs. 42.8%, *p* = 0.103), unplanned readmission (18.5% vs. 16.5%, *p* = 0.515), or 30-day mortality (1.2% vs. 0.5%, *p* = 0.451). However, the MISC cohort had a shorter median length of stay [6 (range 1–55) vs. 7 (3–56); *p* = 0.015] (Table 2).

**Table 1:** Demographics and comorbidities of the propensity-matched cohort

Variables [n (%)]	MISC (*n* = 400)	Open (*n* = 400)	*p* value	SMD
Age, years [Median, (IQR)]	63.50 [26.00, 90.00]	63.00 [29.00, 90.00]	0.800	0.027
BMI, kg/m^2^ [Median, (IQR)]	29.49 [15.98, 62.78]	28.78 [15.60, 58.31]	0.340	0.052
Race			0.996	0.032
Asian	19 (4.8%)	20 (5.0%)		
Black or African American	26 (6.5%)	26 (6.5%)		
White	231 (57.8%)	225 (56.2%)		
Other	121 (30.2%)	126 (31.5%)		
Sex (Male)	278 (69.5%)	289 (72.2%)	0.437	0.061
ASA classification			0.582	0.101
1	3 (0.8%)	7 (1.8%)		
2	128 (32.0%)	126 (31.5%)		
3	248 (62.0%)	250 (62.5%)		
4	21 (5.2%)	17 (4.2%)		
Ascites	0 (0.0%)	0 (0.0%)	1.000	< 0.001
Bleeding disorders	20 (5.0%)	12 (3.0%)	0.206	0.102
Smoking status	59 (14.8%)	65 (16.2%)	0.625	0.041
Diabetes	81 (20.2%)	88 (22.0%)	0.603	0.043
Chronic heart failure (CHF)	1 (0.2%)	5 (1.2%)	0.217	0.116
Chronic obstructive pulmonary disease (COPD)	15 (3.8%)	19 (4.8%)	0.600	0.050
Hypertension requiring medication	207 (51.7%)	212 (53.0%)	0.777	0.025
Steroid use	9 (2.2%)	13 (3.2%)	0.518	0.061
Preoperative chemotherapy	213 (54.2%)	226 (56.6%)	0.520	0.049
Preoperative radiation therapy	215 (54.3%)	221 (55.5%)	0.775	0.025
Preoperative dialysis	1 (0.2%)	1 (0.2%)	1.000	< 0.001
Preoperative transfusion*	4 (1.0%)	3 (0.8%)	1.000	0.027
Preoperative sepsis	4 (1.0%)	4 (1.0%)	1.000	< 0.001
Ventilator dependent	0 (0.0%)	1 (0.2%)	1.000	0.071
Pretreatment clinical cancer stage			0.679	0.062
1	52 (13.0%)	58 (14.5%)		
2	167 (41.8%)	156 (39.0%)		
3	181 (45.2%)	186 (46.5%)		
Tumor location in the rectum			0.650	0.091
Lower third**	171 (42.8%)	181 (45.2%)		
Middle third ***	136 (34.0%)	131 (32.8%)		
Upper third****	59 (14.8%)	49 (12.2%)		
Unknown	34 (8.5%)	39 (9.8%)		
Operative approach			< 0.001	0.702
Laparoscopic	321 (80.2%)	0 (0.0%)		
Open	0 (0.0%)	400 (100.0%)		
Robotic	79 (19.8%)	0 (0.0%)		
Principle procedure			0.537	0.049
Abdominoperineal resection	276 (69.0%)	285 (71.2%)		
Proctectomy	124 (31.0%)	115 (28.7%)		

^*^1 Unit of whole/packed RBCs in 72 h prior to surgery, ** < 5 cm from anal verge, ***5–10 cm from anal verge, **** > 10 cm from anal verge

**Table 2:** Postoperative outcomes of the propensity-matched cohort

Variables [n (%)]	MISC (*n* = 400)	Open (*n* = 400)	*p* value
Any complications	195 (48.8%)	171 (42.8%)	0.103
Superficial incisional SSI	35 (8.8%)	29 (7.2%)	0.515
Deep incisional SSI	6 (1.5%)	9 (2.2%)	0.604
Organ/space SSI	43 (10.8%)	30 (7.5%)	0.140
Wound disruption	8 (2.0%)	8 (2.0%)	1.000
Anastomotic leak	13 (3.2%)	6 (1.5%)	0.162
Ileus	101 (25.2%)	91 (22.8%)	0.728
Acute renal failure	1 (0.2%)	7 (1.8%)	0.069
Sepsis	11 (2.8%)	8 (2.0%)	0.644
Septic shock	6 (1.5%)	6 (1.5%)	1.000
Bleeding transfusions	70 (17.5%)	54 (13.5%)	0.143
Cardiac arrest requiring CPR	2 (0.5%)	1 (0.2%)	1.000
DVT/thrombophlebitis	7 (1.8%)	6 (1.5%)	1.000
Myocardial infarction	0 (0.0%)	7 (1.8%)	0.015
On ventilator > 48 Hours	4 (1.0%)	4 (1.0%)	1.000
Pneumonia	11 (2.8%)	10 (2.5%)	1.000
Pulmonary embolism	3 (0.8%)	4 (1.0%)	1.000
Stroke/CVA	3 (0.8%)	2 (0.5%)	1.000
Urinary tract infection	16 (4.0%)	13 (3.2%)	0.706
Margins (Distal)			0.531
No	11 (2.9%)	11 (2.9%)	
Unknown	13 (3.5%)	20 (5.3%)	
Yes	351 (93.6%)	349 (91.8%)	
Margins (Radial)			0.913
No	41 (10.7%)	38 (9.9%)	
Unknown	12 (3.1%)	13 (3.4%)	
Yes	330 (86.2%)	334 (86.8%)	
Clear distal margin, cm (Median [IQR])	3.00 [0.05, 10.00]	3.00 [0.10, 10.00]	0.455
Clear radial margin, cm (Median [IQR])	1.00 [0.01, 10.00]	1.00 [0.01, 10.00]	0.424
Number of nodes evaluated, n (Median [IQR])	16.00 [1.00, 60.00]	15.00 [0.00, 67.00]	0.060
Length of total hospital stay, days (Median [IQR])	6.00 [1.00, 55.00]	7.00 [3.00, 56.00]	0.015
Unplanned readmission	74 (18.5%)	66 (16.5%)	0.515
Return to OR	23 (5.8%)	22 (5.5%)	1.000
30-day mortality	5 (1.2%)	2 (0.5%)	0.451

In the regression analysis, the MISC group showed no association with an increased risk of postoperative complications (OR = 1.040, 95%CI 0.843–1.280; *p* = 0.716), unplanned readmission (OR = 1.23, 95%CI 0.939–1.620; *p* = 0.132), and 30-day mortality (OR = 1.750, 95%CI 0.648–4750; *p* = 0.269). However, MISC was found to be significantly correlated with a decreased length of stay (β = (−)0.869, 95%CI (−)1.609 − (−)0.129; *p* = 0.021) (Table 3).

**Table 3:** Univariate logistic and linear regression models comparing outcomes of MISC to open

Logistic regression	Odds ratio (MISC/Open)	95% confidence interval	*p* value
Any complication	1.040	0.843–1.280	0.716
30-day mortality	1.750	0.648–4.750	0.269
Unplanned readmission	1.230	0.939–1.620	0.132

Linear regression	Regression Coefficient (MISC/Open)	95% Confidence Interval	*p* value
Postoperative length of stay	− 0.869	− 1.609– − 0.129	0.021

## Discussion

This study compared the postoperative outcomes between patients undergoing minimally invasive surgery converted to open surgery and open surgery for rectal cancer. We found that MISC patients had a shorter median hospital stay (6 vs. 7 days, *p* = 0.015) compared to open surgery patients in the matched cohort. However, there were no significant differences in overall complication rates (48.8% vs. 42.8%, *p* = 0.103), 30-day mortality (1.2% vs. 0.5%, *p* = 0.451), or unplanned readmissions (18.5% vs. 16.5%, *p* = 0.515) between the two groups. Logistic regression analysis further supported these findings, showing no significant differences in complications, mortality, or readmissions, but a significant reduction in postoperative length of stay for MISC patients (*p* = 0.021). Overall, MISC was associated with comparable postoperative outcomes to open surgery, with the added benefit of a shorter hospital stay.

We excluded 5,734 completed MIS from our original study cohort, yielding a conversion rate of 6.61% (Fig. 1). This rate falls on the lower end of the range reported in prior studies, which have reported conversion rates for MIS ranging from 4.1% to 16.6% [6, 9, 11, 19, 20]. The discrepancies in conversion rates may be attributed to multiple factors, including the learning curve of MIS approaches [21, 22]. Our data are from 2016 to 2022, while studies reporting higher conversion rates of 14.3% and 16.6% utilized data from 2009 to 2010 and 2009 to 2012, respectively [9, 11]. Advancements in surgical expertise and technology over time may have contributed to lower conversion rates in our cohort. Robotic surgeries are known to have lower conversion rates than their laparoscopic counterparts, as robotic surgery’s added maneuverability and visualization results in a higher threshold for conversion [7, 10, 23]. The prior studies often reported laparoscopic and robotic procedures separately, while our study pooled data from both platforms, which may further explain the observed discrepancies in conversion rates.

Only two prior studies have directly compared outcomes between MIS converted to open surgery and planned open surgery. In a large retrospective analysis of 65,083 patients with colon cancer using the National Cancer Database (NCDB), Horesh et. al [15] found that conversion from MIS to open surgery was associated with improved overall survival and shorter hospital stays compared to upfront open surgery, without significant differences in 30-day mortality or positive margin rates [15]. Similarly, our study demonstrated that the MISC group had comparable morbidity and 30-day mortality with significantly shorter hospital stays. However, unlike the comparison study, which identified a survival benefit in converted patients, our analysis focused on short-term outcomes and did not find significant differences in morbidity or mortality. This discrepancy may reflect differences in colon and rectal cancers or the technical challenges specific to pelvic surgery [24, 25]. Both studies support the consideration of MIS as an initial approach, even in cases with a higher likelihood of conversion, as it does not appear to compromise patient outcomes and may still offer advantages such as reduced hospital stays. While the comparison study provided robust evidence from a large national database, our findings contribute to the literature by specifically addressing rectal cancer, a population often underrepresented in such analyses.

In a large retrospective analysis of 207,311 colorectal surgery patients from the National Inpatient Sample Database, Masoomi et al. [9] found that converted patients had a significantly lower risk of in-hospital mortality and overall complication rate compared to open cases, but a higher risk of complications such as ileus and abdominal abscess/leak. In contrast, our study did not observe significant differences in 30-day mortality, overall complication rates, or ileus and other specific complications. This discrepancy may be attributed to several factors, including differences in patient populations, surgical techniques, and time periods. Masoomi et al. analyzed the data from 2009 to 2010, when laparoscopic techniques were still evolving and robotic platforms were less common. In contrast, our study spans 2016 to 2022, reflecting increased surgeon experience, and the widespread adoption of robotic surgery, which is associated with lower conversion rates and potentially fewer complications. Additionally, Masoomi et al. included more heterogeneous data that included both colon and rectal surgeries for various indications such as malignancy, diverticulitis, and inflammatory bowel disease. This heterogeneity may have influenced their findings, as rectal surgeries, particularly proctectomies, are known to have higher conversion rates and different complication profiles compared to colon surgeries [25]. In contrast, our study focused specifically on rectal resections for cancer, which may explain the lack of significant differences in complications observed in our cohort.

This study benefits from a large, multicenter, validated surgical dataset, which ensures that the data are reflective of real-world clinical practice and applicable to diverse institutions and patient populations. The large sample size increases the statistical power of the analysis, allowing for robust comparisons between minimally invasive converted to open and open surgery. The use of propensity-score matching helps to reduce selection bias by balancing baseline characteristics between the MISC and open surgery groups, allowing for a more accurate comparison of postoperative outcomes. A key strength of this study is that it is the first to compare MIS converted to open surgeries to open surgeries for rectal cancer, providing valuable insights into the safety and feasibility of attempting MIS in this more technically challenging surgery. Our cases are more homogenous, only including rectal cancer surgeries. Finally, our paper uses a more recent database compared to similar studies.

However, several limitations inherent to large databases, such as the NSQIP, should be acknowledged. The study is constrained by the potential for selection bias and coding inconsistencies, which are common in large-scale datasets and may lead to inaccuracies or type II errors. Furthermore, the database does not capture long-term outcomes, limiting the analysis to 30-day postoperative complications and preventing an assessment of clinically significant long-term results, such as cancer recurrence, survival rates, or quality of life. Important confounders, such as surgical indications, prior surgeries, surgeon experience, and most notably the reason for conversion, are also absent. This could influence outcomes and the interpretation of results. Additionally, the database records only the specialty of a single surgeon per case, omitting the potential impact of collaborative care in complex cases. While the propensity-matched design helps mitigate selection bias, the potential for coding inconsistencies in large databases and the small sample sizes may affect the reliability of the results. Future research should aim to address these limitations by incorporating long-term follow-up, detailed tumor and surgeon-specific data, and a more granular analysis of surgical techniques to provide a more comprehensive understanding of the impact of MISC versus open surgery for rectal cancer.

## Conclusion

Surgeons should consider attempting MIS even in cases with a high likelihood of conversion, as it offers comparable short-term outcomes to open surgery, including similar rates of complications, mortality, and readmissions, while providing the added benefit of reduced hospital stays. Although conversion to open surgery may be necessary in some cases, the initial attempt at MIS does not appear to compromise patient safety and may even yield advantages.

## Supplementary Information

Below is the link to the electronic supplementary material.

Supplementary file1 (DOCX 21 KB)

Supplementary file2 (DOCX 19 KB)
