# Supplementary material for: Minimally invasive converted to open versus upfront open surgeries for rectal cancer: a retrospective cohort study
Source: Surg Endosc. 2025 Jul 8;39(8):5398–405. doi: 10.1007/s00464-025-11958-0 (PMC12287241; doi:10.1007/s00464-025-11958-0)
Supplement: Supplementary file 1 — Supplementary file1 (DOCX 21 KB) [file 464_2025_11958_MOESM1_ESM.docx]

Table S1. Demographics and Comorbidities of the Unmatched Cohort.

| **Variables, [n (%)]** | **MISC (n=406)** | **Open (n=2551)** | **p value** | **SMD** |
| --- | --- | --- | --- | --- |
| Age, years (Median, [IQR]) | 63.50 [26.00, 90.00] | 63.00 [22.00, 90.00] | 0.539 | 0.042 |
| BMI, kg/m^2^ (Median, [IQR]) | 29.49 [15.98, 62.78] | 27.30 [14.48, 64.47] | <0.001 | 0.331 |
| Race |  |  | 0.510 | 0.108 |
| Asian | 19 (4.7%) | 109 (4.3%) |  |  |
| Black or African American | 26 (6.4%) | 157 (6.2%) |  |  |
| White | 232 (57.1%) | 1557 (61.0%) |  |  |
| Other | 129 (31.8%) | 728 (28.5%) |  |  |
| Sex (Male) | 282 (69.5%) | 1533 (60.1%) | <0.001 | 0.197 |
| ASA Classification |  |  | 0.098 | 0.134 |
| 1 | 3 (0.7%) | 25 (1.0%) |  |  |
| 2 | 130 (32.0%) | 663 (26.0%) |  |  |
| 3 | 251 (61.8%) | 1707 (67.0%) |  |  |
| 4 | 22 (5.4%) | 153 (6.0%) |  |  |
| Ascites | 0 (0.0%) | 2 (0.1%) | 1.000 | 0.040 |
| Bleeding Disorders | 20 (4.9%) | 77 (3.0%) | 0.051 | 0.098 |
| Smoking Status | 60 (14.8%) | 503 (19.7%) | 0.017 | 0.131 |
| Diabetes | 83 (20.4%) | 427 (16.7%) | 0.077 | 0.095 |
| Chronic Heart Failure (CHF) | 1 (0.2%) | 21 (0.8%) | 0.348 | 0.079 |
| Chronic Obstructive Pulmonary Disease (COPD) | 15 (3.7%) | 120 (4.7%) | 0.442 | 0.050 |
| Hypertension Requiring Medication | 210 (51.7%) | 1115 (43.7%) | 0.003 | 0.161 |
| Steroid Use | 9 (2.2%) | 95 (3.7%) | 0.147 | 0.089 |
| Preoperative Chemotherapy | 215 (53.9%) | 1435 (56.7%) | 0.302 | 0.057 |
| Preoperative Radiation Therapy | 219 (54.5%) | 1344 (53.2%) | 0.667 | 0.025 |
| Preoperative Dialysis | 1 (0.2%) | 10 (0.4%) | 1.000 | 0.026 |
| Preoperative Transfusion* | 4 (1.0%) | 24 (0.9%) | 0.788 | 0.005 |
| Preoperative Sepsis | 4 (1.0%) | 31 (1.2%) | 1.000 | 0.022 |
| Ventilator Dependent | 0 (0.0%) | 2 (0.1%) | 1.000 | 0.040 |
| Pretreatment Clinical Cancer Stage |  |  | 0.597 | 0.056 |
| 1 | 53 (13.1%) | 370 (14.5%) |  |  |
| 2 | 169 (41.6%) | 1088 (42.6%) |  |  |
| 3 | 184 (45.3%) | 1093 (42.8%) |  |  |
| Tumor Location in the Rectum |  |  | <0.001 | 0.254 |
| Lower third** | 171 (42.1%) | 1245 (48.8%) |  |  |
| Middle third *** | 139 (34.2%) | 698 (27.4%) |  |  |
| Upper third**** | 60 (14.8%) | 258 (10.1%) |  |  |
| Unknown | 36 (8.9%) | 350 (13.7%) |  |  |
| Operative Approach |  |  | <0.001 | 0.695 |
| Laparoscopic | 327 (80.5%) | 0 (0.0%) |  |  |
| Open | 0 (0.0%) | 2551 (100.0%) |  |  |
| Robotic | 79 (19.5%) | 0 (0.0%) |  |  |
| Principle Procedure |  |  | 0.001 | 0.181 |
| Abdominoperineal Resection | 277 (68.2%) | 1946 (76.3%) |  |  |
| Proctectomy | 129 (31.8%) | 605 (23.7%) |  |  |

* 1 Unit of Whole/Packed RBCs in 72 Hours Prior to Surgery

** <5 cm from anal verge

*** 5-10 cm from anal verge

**** >10 cm from anal verge
