# Supplementary material for: Minimally invasive converted to open versus upfront open surgeries for rectal cancer: a retrospective cohort study
Source: Surg Endosc. 2025 Jul 8;39(8):5398–405. doi: 10.1007/s00464-025-11958-0 (PMC12287241; doi:10.1007/s00464-025-11958-0)
Supplement: Supplementary file 2 — Supplementary file2 (DOCX 19 KB) [file 464_2025_11958_MOESM2_ESM.docx]

Table S2. Postoperative Outcomes of the Unmatched Cohort.

| Variables [n (%)] | MISC (n=406) | Open (n=2551) | p value |
| --- | --- | --- | --- |
| Any Complications | 197 (48.5%) | 1213 (47.5%) | 0.748 |
| Superficial Incisional SSI | 36 (8.9%) | 212 (8.3%) | 0.700 |
| Deep Incisional SSI | 6 (1.5%) | 53 (2.1%) | 0.566 |
| Organ/Space SSI | 44 (10.8%) | 203 (8.0%) | 0.054 |
| Wound Disruption | 8 (2.0%) | 84 (3.3%) | 0.169 |
| Anastomotic Leak | 14 (3.4%) | 48 (1.9%) | 0.062 |
| Ileus | 103 (25.4%) | 597 (23.4%) | 0.486 |
| Acute Renal Failure | 1 (0.2%) | 13 (0.5%) | 0.708 |
| Sepsis | 12 (3.0%) | 89 (3.5%) | 0.661 |
| Septic Shock | 6 (1.5%) | 35 (1.4%) | 0.819 |
| Bleeding Transfusions | 70 (17.2%) | 462 (18.1%) | 0.728 |
| Cardiac Arrest Requiring CPR | 2 (0.5%) | 9 (0.4%) | 0.655 |
| DVT/Thrombophlebitis | 7 (1.7%) | 27 (1.1%) | 0.217 |
| Myocardial Infarction | 1 (0.2%) | 25 (1.0%) | 0.245 |
| On Ventilator > 48 Hours | 4 (1.0%) | 16 (0.6%) | 0.343 |
| Pneumonia | 11 (2.7%) | 50 (2.0%) | 0.345 |
| Pulmonary Embolism | 3 (0.7%) | 20 (0.8%) | 1.000 |
| Stroke/CVA | 3 (0.7%) | 6 (0.2%) | 0.115 |
| Urinary Tract Infection | 16 (3.9%) | 106 (4.2%) | 1.000 |
| Margins (Distal) |  |  | 0.386 |
| No | 11 (2.9%) | 66 (2.7%) |  |
| Unknown | 13 (3.4%) | 122 (5.1%) |  |
| Yes | 357 (93.7%) | 2223 (92.2%) |  |
| Margins (Radial) |  |  | 0.338 |
| No | 41 (10.5%) | 259 (10.7%) |  |
| Unknown | 13 (3.3%) | 124 (5.1%) |  |
| Yes | 335 (86.1%) | 2046 (84.2%) |  |
| Clear Distal Margin, cm (Median [IQR]) | 3.00 [0.05, 10.00] | 3.00 [0.01, 10.00] | 0.743 |
| Clear Radial Margin, cm (Median [IQR]) | 1.00 [0.01, 10.00] | 0.80 [0.01, 10.00] | 0.053 |
| Number of Nodes Evaluated, n (Median [IQR]) | 16.00 [1.00, 60.00] | 15.00 [0.00, 100.00] | 0.007 |
| Length of Total Hospital Stay, days (Median [IQR]) | 6.00 [1.00, 55.00] | 7.00 [0.00, 101.00] | 0.010 |
| Unplanned Readmission | 75 (18.5%) | 396 (15.5%) | 0.144 |
| Return to OR | 23 (5.7%) | 163 (6.4%) | 0.660 |
| 30-Day Mortality | 5 (1.2%) | 18 (0.7%) | 0.233 |
